# Safety Assessment of Graphene‐Based Materials

**DOI:** 10.1002/smll.202404570

**Published:** 2025-01-15

**Authors:** Bengt Fadeel, James Baker, Laura Ballerini, Cyrill Bussy, Fabio Candotto Carniel, Mauro Tretiach, Marco Pelin, Tina Buerki‐Thurnherr, Tomi Kanerva, José Maria Navas, Ester Vázquez, Virginia Rodriguez Unamuno, Panja Lehtonen, Mar González, Hubert Rauscher, Juan Riego Sintes, Kostas Kostarelos, Alberto Bianco, Maurizio Prato

**Affiliations:** ^1^ Institute of Environmental Medicine Karolinska Institutet Stockholm 17177 Sweden; ^2^ TEMAS Solutions (TEMASOL) Hausen 5212 Switzerland; ^3^ International School for Advanced Studies (SISSA) Trieste 34136 Italy; ^4^ Centre for Nanotechnology in Medicine School of Biological Sciences Faculty of Biology Medicine & Health and National Graphene Institute Manchester M13 9PT, and National Graphene Institute University of Manchester Manchester M13 9PL UK; ^5^ Department of Life Sciences University of Trieste Trieste 34127 Italy; ^6^ Laboratory for Particles‐Biology Interactions Swiss Federal Laboratories for Materials Science and Technology (EMPA) St. Gallen 9014 Switzerland; ^7^ Finnish Institute of Occupational Health (FIOH) Helsinki 00032 Finland; ^8^ Instituto Nacional de Investigación y Tecnología Agraria y Alimentaria (INIA) Consejo Superior de Investigaciones Científicas (CSIC) Madrid 28040 Spain; ^9^ Instituto Regional de Investigación Científica Aplicada (IRICA) and Facultad de Ciencias y Tecnologías Químicas Universidad de Castilla‐La Mancha Ciudad Real 13071 Spain; ^10^ European Chemicals Agency (ECHA) Helsinki 00150 Finland; ^11^ Organisation for Economic Co‐operation and Development (OECD) Paris 75016 France; ^12^ European Commission Joint Research Centre (JRC) Ispra 21027 Italy; ^13^ Catalan Institute of Nanoscience and Nanotechnology (ICN2) CSIC and BIST Campus UAB (Universitat Autònoma de Barcelona) Bellaterra 08193 Spain; ^14^ Institució Catalana de Recerca i Estudis Avançats (ICREA) Barcelona 08010 Spain; ^15^ CNRS Immunology, Immunopathology and Therapeutic Chemistry UPR 3572 University of Strasbourg, ISIS Strasbourg 67000 France; ^16^ Center for Cooperative Research in Biomaterials (CIC biomaGUNE) Basque Research and Technology Alliance (BRTA) San Sebastián 20014 Spain; ^17^ Ikerbasque Basque Foundation for Science Bilbao 48009 Spain; ^18^ Department of Chemical and Pharmaceutical Sciences University of Trieste Trieste 34127 Italy

**Keywords:** advanced materials, graphene, safety assessment, test guidelines

## Abstract

Graphene is the first 2D atomic crystal, and its isolation heralded a new era in materials science with the emergence of several other atomically thin materials displaying multifunctional properties. The safety assessment of new materials is often something of an afterthought, but in the case of graphene, the initial isolation and characterization of the material was soon followed by the assessment of its potential impact on living systems. The Graphene Flagship project addressed the health and environmental aspects of graphene and other 2D materials, providing an instructive lesson in interdisciplinarity – from materials science to biology. Here, the outcomes of the toxicological and ecotoxicological studies performed on graphene and its derivatives, and the key lessons learned from this decade‐long journey, are highlighted.

## Graphical abstract

This perspective provides an overview of the main achievements of the Health & Environment work package of the Graphene Flagship (2013–2023), a pan‐European project funded by the European Commission. The project encompassed approximately 170 academic and industrial research partners and 90 associated members.

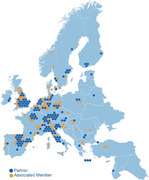

## 1 Graphene Flagship–A 10‐Year Journey

Graphene, a 2D atomic crystal, was first described in a seminal paper by Geim and colleagues in 2004.^[^ ^1^ ^]^ Now, 20 years after the “Big Bang”, the universe of 2D materials is ever‐expanding. Currently, the most studied 2D materials apart from graphene‐based materials (GBMs) are hexagonal boron nitride (hBN), transition metal dichalcogenides (TMDs), and transition metal carbides and nitrides (MXenes) including titanium carbide (Ti_3_C_2_) MXenes and many others based on combinations of various transition metals.^[^ ^2^ ^]^ These materials are being considered for a plethora of applications in electronics and optoelectronics, electrocatalysis, and energy storage, and as components in composites, or as absorbents (for environmental remediation), as well as biomedical applications including drug delivery, photodynamic/photothermal therapy, medical imaging, and wearable and implantable devices.^[^ ^3^, ^4^ ^]^ The discovery of 2D materials has thus provided a powerful toolbox for material scientists. This raises another challenge, namely, how to develop and use these materials safely.^[^ ^5^ ^]^

The Graphene Flagship (2013–2023) (www.graphene‐flagship.eu) is the largest‐ever research initiative in the EU. The project involved more than 170 academic and industrial partners along with 90 partners belonging to associated projects funded by the member states and has spawned more than 5000 scientific publications and helped to launch 20 spin‐off companies. Indeed, one of the key goals has been the creation of a commercial ecosystem for graphene and other 2D materials in the EU, and safety assessment is integral to this effort. To this end, an important element has been the work package (WP) devoted to Health and Environment, a “project within a project” encompassing one dozen universities and other partner institutes. The impetus for the present perspective was provided by a roundtable discussion on human health and the environment held at the University of Trieste in September 2023. In addition to the WP members, external experts from the European Chemicals Agency (ECHA), the Joint Research Centre (JRC) of the European Commission, and the Organisation for Economic Co‐operation and Development (OECD) also participated. This perspective aims to capture the vibrant discussions that took place at the roundtable. We believe that the lessons learned in the Graphene Flagship are relevant also for the safe production and use of other advanced materials.

The present perspective is thus focused on the work conducted during the decade‐long Graphene Flagship, but we certainly acknowledge that seminal work has also been performed beyond the latter project. To gain a better picture of the graphene publication landscape during the period 2004–2024, we conducted a bibliometric survey using Web of Science 2023 of Clarivate Analytics (UK) Ltd. The specific search terms can be found in the Supporting Information of the present article. The results showed that China (Asia) accounted for the lion's share of all publications, followed by Europe, and North America (Figure S1, Supporting Information). We also performed a network analysis of co‐publishing universities and other research institutes in Europe to gain further insight regarding the key players during the period 2008–2024. The results, depicted in Figure S2 (Supporting Information), illustrate the connectivity between several major research organizations in Europe, including the CNRS in France, and the CNR in Italy, and other major players, such as the University of Manchester (the home of graphene research).

## 2 Not One Material but a Class of Materials

Graphene should not be viewed as a single type of material but as a class of materials. This seems trivial, but it is important to keep in mind, as is the fact that nanomaterial interactions with biological systems depend not only on the nanomaterial and its properties but also on the (dynamic) biological environment. The potential applications for graphene and other atomically thin materials are manifold, but the biomedical use of nanomaterials including drug delivery, imaging, or both, is often seen as the Holy Grail. However, as Novoselov et al.^[^ ^6^ ^]^ noted in an essay in 2012, “Before graphene can fulfill its promise in the biomedical area, we must understand its biodistribution, biocompatibility, and acute and chronic toxicity [and] the outcome is likely to vary with size, morphology, and chemical structure.” These remarks are prescient, as studies conducted over the past decade have confirmed that the biological impact of graphene and its derivatives varies depending on the material properties.^[^ ^7^, ^8^ ^]^ In other words, size matters, in the sense that the lateral dimensions and thickness or number of layers of the nanosheets are important determinants of their impact on living systems. The chemical composition also matters; pristine graphene is not the same as graphene oxide (GO) or reduced graphene oxide (rGO). Indeed, if we have learned anything from the past decade or more of nanosafety research, it is that surface properties of nanomaterials are important. Graphene and GO differ in terms of their hydrophobicity/hydrophilicity: the surface of pristine graphene is hydrophobic while the surface of GO is composed of hydrophobic islands with hydrophilic regions showing various degrees of reactivity.^[^ ^9^ ^]^ This has significant consequences for the dispersibility of the materials in aqueous media as well as for their biological effects.^[^ ^7^, ^8^ ^]^

The Graphene Flagship published a “science and technology roadmap” in 2015.^[^ ^10^ ^]^ The authors cautioned that “to avoid the generalization often found for other carbon‐based nanomaterials, such as carbon nanotubes, it is necessary to take into consideration the great variability of the materials tested.” There has been a tendency to group nanomaterials, especially carbon‐based nanomaterials, into one material category, and some have argued that “graphene may develop into [a] fiber‐shaped material, since the flakes or sheets may coil after production”.^[^ ^11^ ^]^ However, this statement is misleading as it does not take into account the biological softness (or rigidity) of the materials.^[^ ^12^ ^]^ The lateral dimensions may play a role, but invoking the pathogenic fiber paradigm is not helpful as graphene and its derivatives are known to be susceptible to degradation both by bacterial and human cells.^[^ ^13^, ^14^ ^]^ Moreover, 10 years ago, “graphene” was often used as a catch‐all phrase for the whole class of materials. Li et al.^[^ ^15^ ^]^ suggested that graphene microsheets physically disrupted the cytoskeletal organization of mammalian cells and provided evidence that graphene sheets entered the cells edge‐first. This study, which has been widely cited, thus gives the impression that graphene causes physical damage to cells by piercing the cell membrane. However, subsequent studies performed in the Graphene Flagship have shown that thin GO sheets of varying lateral dimensions are readily internalized by primary human macrophages in the absence of toxicity.^[^ ^16^ ^]^ It is important to point out that the GO sheets used in the latter study were free from endotoxin, as the presence of endotoxin (a bacterial contaminant) may otherwise confound the results.

The antimicrobial activity of graphene and its derivatives also merits attention. Both physical (edge‐dependent) and chemical mechanisms of bacterial killing may come into play. Lu et al. demonstrated that the vertical alignment of GO sheets yielded an enhanced antibacterial effect,^[^ ^17^ ^]^ while graphene flakes grown by a plasma‐enhanced chemical vapor deposition process were found to prevent biofilm formation.^[^ ^18^ ^]^ Moreover, by controlling the height of the flakes, the toxicity toward mammalian cells could be avoided. Graphene‐based polymeric composites and composites formed by combining GBMs with metals or metal oxides are also being studied for their antibacterial activity.^[^ ^19^ ^]^ Additionally, 3D graphene architectures (foams) are being explored with respect to environmental remediation. For instance, so‐called spongy graphene prepared by reducing a colloidal suspension of GO followed by molding was proposed as a sorbent for commercial petroleum products and organic solvents.^[^ ^20^ ^]^ Safety assessment is required to maximize such effects while safeguarding human health and the environment. The members of the WP on Health and Environment devised a classification framework for GBMs to promote the safety assessment of these materials.^[^ ^9^ ^]^ This framework, published in 2014, considers the number of layers, the average lateral size, and the carbon‐to‐oxygen ratio as the three main properties that should be defined when assessing biological effects. Subsequent studies performed in vitro and in vivo have confirmed that this is a useful framework.^[^ ^7^, ^8^ ^]^ However, it is important to consider that the functionalization of graphene^[^ ^21^ ^]^ is an additional parameter that may influence the subsequent biological profile of GBMs.^[^ ^22^ ^]^ The adsorbed surface layer of proteins and other biomolecules (aka bio‐corona) also needs to be taken into account when addressing the potential hazard of GBMs as well as other 2D materials.^[^ ^23^, ^24^ ^]^ Indeed, recent studies have shown that tailoring the surface chemistry of GBMs could regulate the formation of the bio‐corona on the surface,^[^ ^25^ ^]^ and others have shown that the biocompatibility of GO is linked to the bio‐corona composition.^[^ ^26^ ^]^ The question is to what extent such classification frameworks can be applied to 2D materials beyond graphene. The thickness and lateral dimensions may play a role, but the chemical composition is also a key determinant of the biological effects of “post‐graphene” materials, as is the rate of dissolution of the material.^[^ ^27^ ^]^ The latter parameter may be of particular importance for the transition metal‐based 2D materials.^[^ ^28^, ^29^ ^]^ Importantly, while 2D materials are relatively new entities, the biological (and toxicological) effects of metal ions have been widely studied both in relation to human health and the environment.^[^ ^30^ ^]^ Surface functionalization (passivation) can modulate the toxicological profile of 2D materials, as shown for phosphorene^[^ ^31^ ^]^ and TMDs.^[^ ^32^ ^]^ The latter study is instructive since it sheds light on another important feature of 2D materials, namely, surface vacancies. TMDs may contain a number of different structural defects in their crystal lattices which may alter the physicochemical as well as the biological properties.^[^ ^32^ ^]^ MXenes also display vacancies but there are no systematic studies to date regarding the toxicological implications of such surface defects. Nevertheless, studies on 2D materials, along with previous studies performed on a range of conventional (spherical and fiber‐shaped) nanomaterials,^[^ ^33^ ^]^ have confirmed the importance of surface properties in determining the hazard potential. However, there is no single material property that can be queried to assess the toxicity of nanomaterials. Therefore, careful material characterization^[^ ^34^ ^]^ is required to unravel the impact of 2D materials.

## 3 Lessons Learned: from Mice to Humans

The aim of the present perspective is not to provide an exhaustive survey of the work performed in the Graphene Flagship. To this end, we refer the reader to other recent publications.^[^ ^7^, ^8^ ^]^ Instead, we will highlight some of the key take‐home messages. Overall, evidence for excessive or alarming toxicity could not be found for any of the tested materials. However, this is a statement that needs to be qualified in more ways than one. First, toxicity is related to the dose, and the duration of the exposure is also important. Most toxicological studies of 2D materials have been conducted using high doses (often excessively high in relation to real‐world exposure scenarios) with emphasis on acute effects (typically 24–48 h). This may be useful if the objective is to investigate the hazard potential or a specific mechanism of toxicity. However, data from such studies may be of limited value in terms of understanding the risk of adverse health effects in the occupational setting or the risk of environmental impacts of the materials.^[^ ^35^ ^]^ Indeed, it is worth noting that the impact of GO on human lung cells is markedly different following acute exposure (48 h) to a single dose as compared to repeated exposure twice per week for 4 weeks at the same cumulative dose.^[^ ^36^ ^]^ Furthermore, most if not all toxicological studies have focused on as‐produced materials, but it is also important to understand whether the hazard potential of 2D materials varies along the life cycle of the material or product. Using a panel of in vitro models representative of different target organs, a recent study conducted by members of the Graphene Flagship showed a negligible impact of rGO‐enforced composites.^[^ ^37^ ^]^ Moreover, the abraded composites induced a modest and transient pulmonary inflammation in mice which was resolved 28 days after exposure. Similarly, a recent follow‐up study of hBN‐reinforced composites subjected to weathering showed minimal effects on human lung and skin cells.^[^ ^38^ ^]^ Additionally, other investigators have shown that GO that had been aged over time in water versus GO that had undergone accelerated aging through sonication remained cytocompatible.^[^ ^39^ ^]^

One important lesson is that high‐quality toxicological investigations require high‐quality (i.e., well‐defined) materials, especially if the objective is to assign the observed biological effects to specific material properties.^[^ ^9^ ^]^ To this end, considerable efforts have been devoted to the synthesis and characterization of endotoxin‐free GO sheets with controlled lateral dimensions.^[^ ^40^ ^]^ GO sheets were thus produced using a modified Hummers’ method, and physicochemical properties of the GO samples were exhaustively characterized using a battery of techniques including atomic force microscopy, transmission electron microscopy, Fourier‐transformed infra‐red spectroscopy, X‐ray photoelectron spectroscopy, and Raman spectroscopy. Using such GO sheets, several in vitro and in vivo studies were conducted. For instance, radiolabeled GO sheets were investigated with respect to their pharmacokinetic profiles upon i.v. administration in mice.^[^ ^41^ ^]^ The results revealed that large, small, and ultra‐small GO sheets were all sequestered by the spleen and liver. Moreover, a significant accumulation of large GO sheets was observed in the lungs, likely in pulmonary capillaries. Interestingly, extensive urinary excretion of all three GO materials was documented; however, the rate of excretion was affected by the lateral size of the 2D materials. Furthermore, additional studies using GO sheets of varying lateral dimensions revealed size‐dependent effects following single or repeated pulmonary exposure in mice.^[^ ^42^, ^43^ ^]^ Hence, tissue granulomas (aggregations of macrophages and other immune cells) were seen only for micrometric GO sheets. The authors also found that only multi‐walled carbon nanotubes (MWCNTs) triggered fibrosis, an irreversible scarring of the lungs, while this was not observed for GO sheets irrespective of the lateral dimensions.^[^ ^42^ ^]^ Again, it is important to consider that large GO sheets remain flexible whereas MWCNTs behave like rigid needles.^[^ ^43^ ^]^

**Figure 1:**
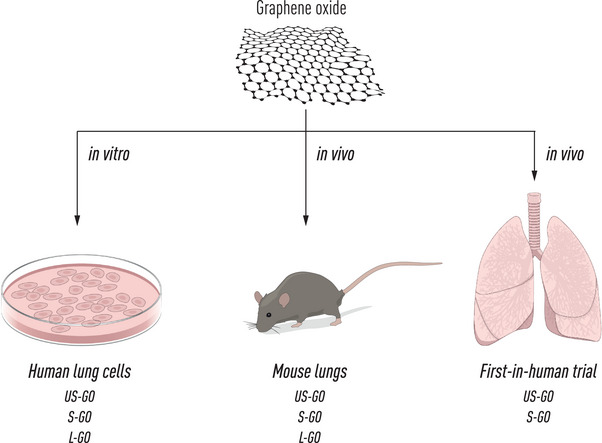
From cell culture to healthy volunteers. Studies conducted in the Graphene Flagship have shown that graphene oxide (GO) elicited size‐dependent effects in human lung cells. Similarly, studies using GO sheets of varying lateral dimensions revealed size‐dependent effects following pulmonary exposure in vivo insofar as large (L) GO but not small (S) or ultrasmall (US) GO provoked tissue granulomas in the lungs of mice. Finally, armed with this knowledge, a recent first‐in‐human controlled inhalation study was conducted using purified S‐GO and US‐GO, and exposure to US‐GO was found to be well tolerated, with no acute adverse effects.^[^ ^44^ ^]^

Inhalation is the primary route of unintentional (occupational) exposure to nanomaterials, and it also represents an important potential route of administration for nanomedicines. However, there have been no human inhalation studies on GO or, for that matter, any other 2D materials. To address this knowledge gap, a recent study was conducted on healthy human volunteers using thin, purified (endotoxin‐free) GO sheets with small lateral dimensions (**Figure** **1**). To this end, 14 volunteers were enrolled, and the potential impact of GO sheets on cardiorespiratory endpoints following a single dose was investigated. No signs of adverse effects were observed.^[^ ^44^ ^]^ This work has demonstrated the feasibility of conducting carefully controlled human inhalation studies of GO, but the authors acknowledged some limitations: only a single dose was tested, and the follow‐up of the volunteers was limited to a 6‐h period after the start of the exposure.^[^ ^44^ ^]^

## 4 Probing the Biological Interactions of Graphene

**Figure 2:**
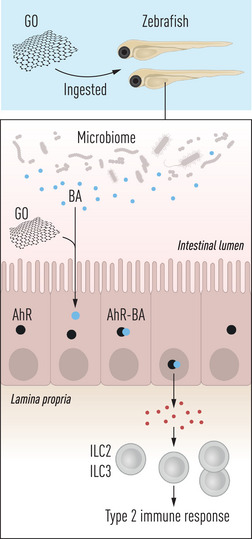
New frontier in the safety assessment of graphene‐based materials. Using zebrafish as a model, Peng et al.^[^ ^52^ ^]^ showed that GO elicited AhR‐dependent induction of type 2 immune cells when combined with the microbial short‐chain fatty acid, butyric acid (BA). The AhR‐dependent signal responsible for the induction and/or recruitment of innate lymphoid cell (ILC)‐like cells remains to be identified. Nevertheless, these studies revealed for the first time that a 2D material (such as GO) can modulate the crosstalk between the microbiome and the immune system.

Comprehensive in vitro (cell‐based) studies performed in the Graphene Flagship have shown that GBMs are non‐cytotoxic for human immune cells including macrophages, neutrophils, dendritic cells, and others, whereas other 2D materials such as MoS_2_ and WS_2_ were found to trigger very modest effects.^[^ ^45^, ^46^, ^47^, ^48^, ^49^ ^]^ This is in contrast to other metal‐based nanomaterials which may elicit strong cytotoxicity toward human macrophages.^[^ ^50^ ^]^ However, this does not mean that 2D materials are completely innocuous as they could also influence cellular function in the absence of overt cell death.^[^ ^51^ ^]^ Therefore, it is important to go beyond dead‐or‐alive assays and study cell function, e.g., cytokine secretion, antigen presentation, and so on. Furthermore, it is conceivable that 2D materials may indirectly impart their immunomodulatory effects. For instance, GO provoked immune responses in zebrafish via the gut microbiome and its metabolites.^[^ ^52^ ^]^ Specifically, GO endowed with a bio‐corona of butyric acid (butyrate) elicited an aryl hydrocarbon (Ah) receptor‐dependent response in the gut leading to the induction of a type 2 immune response, similar to the innate immune response elicited by parasitic infections (**Figure** **2**). The latter study underscores the importance of applying relevant in vivo models to gain a deeper appreciation of the complex interactions between 2D materials and biological systems. Notwithstanding, the overall conclusion from all of these studies is that GBMs do not elicit remarkable toxicities in vitro or in vivo, with the caveat that not all GBMs are alike. Further studies are needed with respect to other post‐graphene materials including MXenes.^[^ ^27^ ^]^

**Figure 3:**
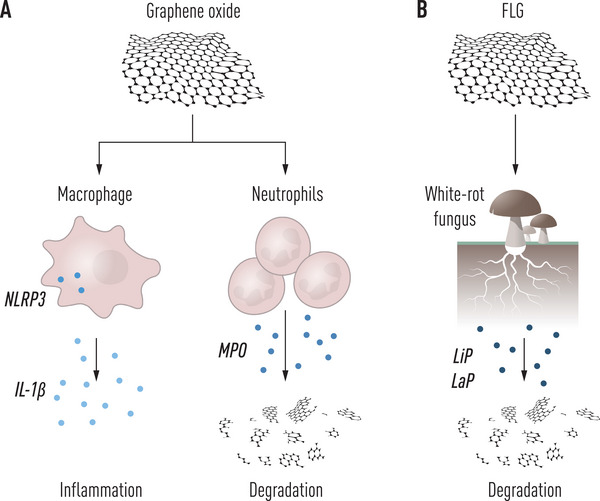
From inflammation to degradation. a) Recent studies in the Graphene Flagship have disclosed that graphene oxide (GO) is capable of triggering the cytosolic NLRP3 inflammasome complex in macrophages leading to the secretion of the proinflammatory cytokine IL‐1β. On the other hand, GO is also susceptible to degradation by myeloperoxidase (MPO) which is abundantly expressed in neutrophils. b) Furthermore, few‐layer graphene (FLG) is oxidized to a GO‐like material by lignin peroxidase (LiP) and/or laccase/peroxidase (LaP) over a period of several months, implying that graphene is also prone to degradation in terrestrial ecosystems.^[^ ^64^ ^]^

One of the key discoveries in the Graphene Flagship is the fact that GBMs are susceptible to degradation in cells and living organisms as well as in the environment (**Figure** **3**), and this may finally lay to rest any concerns related to asbestos‐like properties of this class of materials.^[^ ^53^ ^]^ Hence, GO can be “digested” by myeloperoxidase (MPO), an enzyme that is abundantly expressed in neutrophils.^[^ ^54^, ^55^ ^]^ MPO produces hypochlorite, a very harsh oxidant that is normally involved in the microbicidal activities of these cells. FLG as well as graphene quantum dots and graphene nanoribbons are also susceptible to degradation albeit to a lesser extent than GO,^[^ ^56^, ^57^, ^58^ ^]^ and evidence has been provided for the partial degradation of hBN (aka white graphene).^[^ ^59^ ^]^ Importantly, recent studies have confirmed that biodegradation of GBMs can also occur in vivo.^[^ ^60^ ^]^ Loret et al.^[^ ^61^ ^]^ monitored material elimination from the lungs of mice by using Raman spectroscopy following a single exposure to FLG or GO of varying lateral dimensions. Notably, while all tested materials could be identified in the lungs on day 1 post‐exposure, only the large GO sheets remained in the lungs on day 28. Furthermore, while neutrophil influx was noted on day 1, alveolar macrophages appeared to play a dominant role in the sequestration and elimination of the GBMs.^[^ ^61^ ^]^ Moreover, evidence for biodegradation of GO in the marginal zone of the spleen over a period of 9 months was provided in a model of i.v. administration of GO.^[^ ^62^ ^]^ Other investigators suggested that carbon‐based nanomaterials including GO might serve as a novel carbon source for the gut microbiota in mice.^[^ ^63^ ^]^ However, no differences were noted with respect to the biodegradation of GO in conventional and germ‐free zebrafish (Fadeel, et al., unpublished observations). Studies performed in the Graphene Flagship have also shown that FLG is susceptible to degradation by certain fungi present in soil.^[^ ^64^, ^65^ ^]^

Large‐scale computer simulations have provided valuable insights with respect to the biological interactions of graphene and its derivatives.^[^ ^66^ ^]^ Based on molecular dynamics (MD) simulations, Tu et al. postulated that graphene could induce degradation of the membranes of *Escherichia coli* through a lipid extraction mechanism.^[^ ^67^ ^]^ Furthermore, Mao et al. identified the degree of oxidization and lateral size of graphene sheets as key drivers of membrane perturbation,^[^ ^68^ ^]^ while other investigators provided evidence that GO nanosheets could retard cancer cell migration through the disruption of intracellular actin filaments.^[^ ^69^ ^]^ In the latter study, large‐scale all‐atom MD simulations revealed the interactions between GO nanosheets and actin filaments in molecular detail. Similar modeling approaches have also been applied to other 2D materials. In a recent study, the interaction of hBN with cell membranes was elucidated by combining MD simulations with experimental approaches.^[^ ^70^ ^]^ The simulations revealed that hBN could penetrate the lipid bilayer and form a transmembrane water channel along its exposed polar edges, and it was hypothesized that this could contribute to lysosomal membrane permeabilization and cell death.^[^ ^70^ ^]^ It is also noted that the material properties responsible for the cytotoxic behavior of 2D materials could potentially be predicted using machine learning (ML) approaches.^[^ ^71^ ^]^ However, the success of ML models relies heavily on the quality and quantity of available data.

## 5 The Need for Harmonization and Standardization

There is an overall consensus that while existing regulatory frameworks apply to nanomaterials, risk assessment should consider the specific properties of these materials.^[^ ^72^ ^]^ This may require adaptation of the test methods which are normally applied for the assessment of potential effects of chemicals. Over the last several years, considerable efforts have focused on adapting existing and/or developing new OECD test guidelines (TGs) and guidance documents (GDs) for nanomaterials.^[^ ^73^ ^]^ Much of this work is linked to the so‐called Malta Initiative which originated during the Maltese EU Council Presidency in 2017 when Germany approached the EU Directorate General for Research and Innovation (DG RTD) to request political and financial support to develop and amend TGs and GDs to ensure that regulatory requirements are addressed with respect to nanomaterials. The Malta Initiative brings together EU member states, the European Commission, and ECHA, as well as industry and other institutions. The EU‐funded project H2020‐NanoHarmony published a white paper in October 2023 with recommendations for streamlining the process of adapting and developing OECD TGs (the report can be downloaded at: www.nanosafetycluster.eu). Members of the Graphene Flagship have addressed the applicability of OECD TGs with respect to GBMs (refer to Figure S3, Supporting Information for an overview), and suggestions for improvements of some TGs were provided.^[^ ^74^, ^75^ ^]^ Other investigators have also highlighted the importance of adapting test methods for GBMs.^[^ ^76^ ^]^

Since 2013, the JRC has hosted a repository of industrially manufactured nanomaterials.^[^ ^77^ ^]^ These materials were initially tested under the auspices of the OECD Testing Programme, and they have since been applied in several EU‐funded research projects including FP7‐NANOREG, a project comprised of over 85 institutional partners from EU member states and other collaborating countries.^[^ ^78^ ^]^ The materials in the nanomaterials repository may be viewed as “benchmark” materials,^[^ ^79^ ^]^ and their wide adoption facilitates the generation of comparable and reliable experimental results across different laboratories, ultimately supporting the development of OECD TGs.^[^ ^77^ ^]^ We submit that there is also a need for benchmark materials to support the development of harmonized test protocols for 2D materials. This means that standardized protocols for the preparation of working solutions (dispersions) of such materials are needed. Flagship partners have addressed this challenge, and a protocol for the preparation of aqueous suspensions of graphene – a strongly hydrophobic material – has been developed.^[^ ^80^ ^]^ Furthermore, it is noted that while GBMs produced at laboratory scale tend to be well‐controlled, concerns have been raised that commercial materials nominally labeled as “graphene” are heterogeneous and may also contain impurities.^[^ ^81^ ^]^ There is thus a need for validated and harmonized test methods, as well as a need for benchmark or reference materials. There is also a need for a harmonized knowledge infrastructure in nanosafety,^[^ ^82^ ^]^ including databases that are compliant with the so‐called F.A.I.R. principles for data management.^[^ ^83^ ^]^ The European Union Observatory for Nanomaterials (EUON) hosted by ECHA (www.euon.echa.europa.eu) serves as a useful example of a federated repository of knowledge.

The EU Chemicals Strategy for Sustainability (CSS) action plan foresees the development of a framework to define “safe‐and‐sustainable‐by‐design” (SSbD) criteria for chemicals and materials. The JRC has recently developed and published a framework for the definition of possible SSbD criteria and implementation mechanisms.^[^ ^84^ ^]^ The SSbD concept goes beyond the hazard assessment of chemicals and takes a holistic approach by integrating the safety, circularity, and functionality of chemicals, materials, products, and processes throughout their life cycle. Overall sustainability is ensured by minimizing the environmental footprint of chemicals and materials.^[^ ^85^ ^]^ Understanding the mechanism of toxicity of 2D materials and other nanomaterials, and linking this to specific material properties, is key to this endeavor, as this would enable material scientists to “design out” the offending properties. Indeed, as previously pointed out,^[^ ^86^ ^]^ “mechanistic models of toxicity could be the ultimate weapon against uncertainty if they provide a […] link between specific nanomaterial features and a particular biological response.” Thus, while there is a need for harmonized test protocols, fundamental research is also needed.

## 6 Outlook: A Perspective on Advanced Materials

The Graphene Flagship project has come to an end, but the voyage continues in the form of an armada of smaller and separately funded research projects. Meanwhile, there is a strong drive toward so‐called advanced materials, i.e., materials purposefully engineered to exhibit properties (including but not restricted to size) that confer unique or superior performance relative to conventional materials,^[^ ^87^ ^]^ as evidenced by the Materials 2030 Roadmap, and other similar initiatives. Nanotechnology has paved the way for these developments, and we must learn from past experiences in terms of dealing with novel materials. Indeed, responsible development and interdisciplinarity have been a lodestar for nanotechnology,^[^ ^88^ ^]^ and international cooperation and the sharing of protocols and data are important keys to success.^[^ ^89^ ^]^

Nanotechnology is one of the technologies that could enable a sustainable future. Thus, while every effort should be made to ensure that nanomaterials including 2D materials are safe, it is important to not lose sight of the fact that the materials themselves may offer solutions to environmental problems, for instance, through the remediation of environmental contaminants.

The OECD Working Party on Manufactured Nanomaterials (WPMN) serves as a global forum for discussions on the safety of nanomaterials and other advanced materials from a policy and regulatory perspective. The Early Awareness and Action System for Advanced Materials (Early4AdMa), published in September 2023, is a pre‐regulatory and anticipatory risk governance approach for advanced materials. Early4AdMa helps users (regulators) identify potential issues related to safety, sustainability, and/or regulatory needs at the early stages of development or use of advanced materials.^[^ ^90^ ^]^ Case studies are in progress to assess the applicability of the Early4AdMa approach; these case studies encompass 2D materials such as MXenes. Thus, advanced materials are not a thing of the future, but something that has been under investigation from a health and environmental perspective for some time, due in no small part to the Graphene Flagship. Indeed, it is our firm conviction that the latter project may serve as a blueprint for the safety assessment of emerging 2D materials and other advanced materials.

## Conflict of Interest

The authors declare no conflict of interest.

## Supporting information



Supporting Information
