# Supplementary material for: Safety Assessment of Graphene‐Based Materials
Source: Small. 2025 Jan 15;21(7):2404570. doi: 10.1002/smll.202404570 (PMC11840464; doi:10.1002/smll.202404570)
Supplement: Supplementary file 1 — Supporting Information [file SMLL-21-2404570-s001.pdf]

NANO · MICRO
small

Supporting Information

for *Small*, DOI 10.1002/smll.202404570

Safety Assessment of Graphene-Based Materials

Bengt Fadeel, James Baker, Laura Ballerini, Cyrill Bussy, Fabio Candotto Carniel, Mauro Tretiach, Marco Pelin, Tina Buerki-Thurnherr, Tomi Kanerva, José Maria Navas, Ester Vázquez, Virginia Rodriguez Unamuno, Panja Lehtonen, Mar González, Hubert Rauscher, Juan Riego Sintes, Kostas Kostarelos, Alberto Bianco* and Maurizio Prato**

Supporting Information

Safety assessment of graphene-based materials

Fadeel B, Baker J, Ballerini L, Bussy C, Candotto Carniel F, Tretiach M, Pelin M, Buerki-Thurnherr T, Kanerva T, Navas JM, Vázquez E, Rodriguez Unamuno V, Lehtonen P, González M, Rauscher H, Riego Sintes J, Kostarelos K, Bianco A, and Prato M

Contents:

Method: bibliometric analysis (definition of search terms) (period: 2004-2024)

Supplementary Figure S1 (all continents: number of publications 2004-2024)

Supplementary Figure S2 (network of co-publishing organizations in Europe)

Supplementary Figure S3 (summary of OECD test guideline (TG) evaluation)

Bibliometric analysis

Data extraction: 2024-08-30. Publication years: 2004-2024. Total number of records: 15014. No manual curation was performed apart from the fact that retracted articles were removed prior to further analysis. This, therefore, means that articles including the word ”toxicity” in the title or abstract may or may not include experimental results pertaining to toxicity. Nevertheless, we decided to cast a wide net to ensure that all the relevant articles were included. Note that for the network analysis of co-publishing organizations, the period was 2008-2024 (however, only a small number of relevant articles were published between 2004-2008). For the latter analysis, the origin of each article was determined on the basis of the affiliation of the first author. Data analysis performed by Karolinska Institutet University Library (Stockholm). Data were derived from © Web of Science 2023 of Clarivate Analytics (UK) Ltd.

Search criteria used:

"Exact search" used.

(
    (TI=(((2d or “two dimensional” or “two-dimensional”) NEAR/2 material*))) OR AB=(((2d or “two dimensional” or “two-dimensional”) NEAR/2 material*))
    OR (TI=(graphene*)) OR AB=(graphene*)
)
AND
(
    TS=(toxic* OR ecotoxic* OR cytotoxic* OR genotoxic* OR nanotoxic*)
    OR TS=(biocompatib* OR safety)
    OR (TI=(((environment* or human) NEAR/3 (assessment or hazard or exposure* or risk)))) OR AB=(((environment* or human) NEAR/3 (assessment or hazard or exposure* or risk)))
)
AND PY=2004-2024
AND DT=(Article OR Review)

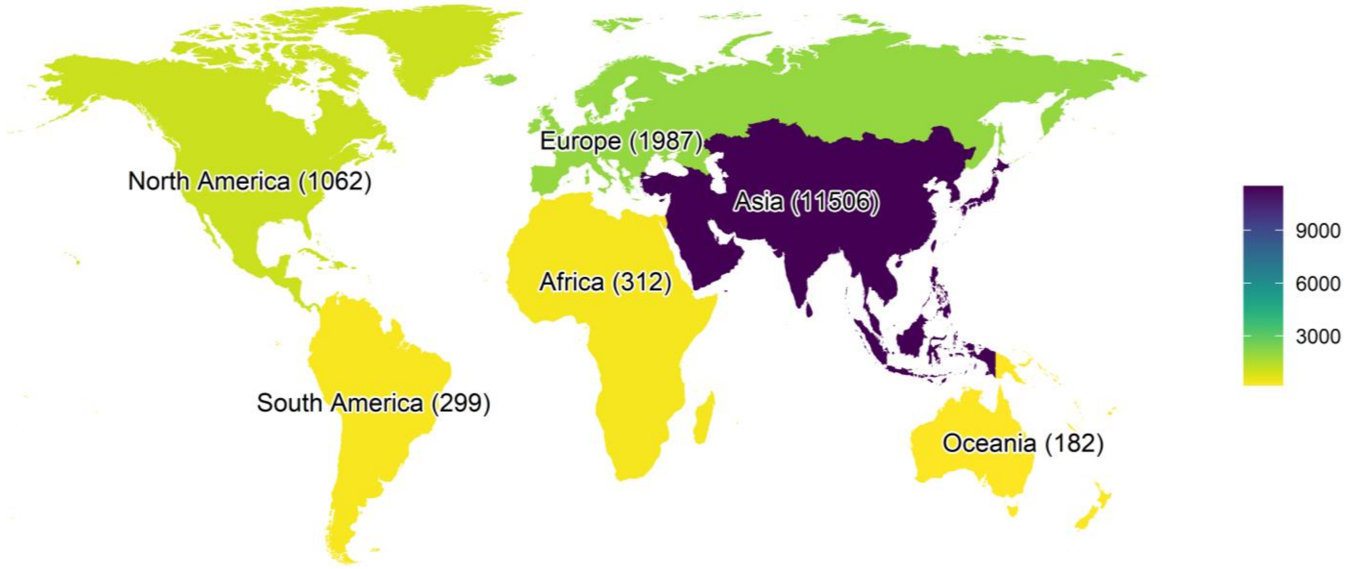

Figure S1. Total number of publications per continent during the period 2004-2024. The search terms are defined in the section labeled ”bibliometric analysis” and correspond to articles related to graphene and biocompatibility/safety/(eco)toxicity/human health and/or environment. The results were extracted from © Web of Science 2023 of Clarivate Analytics (UK) Ltd on August 30, 2024.

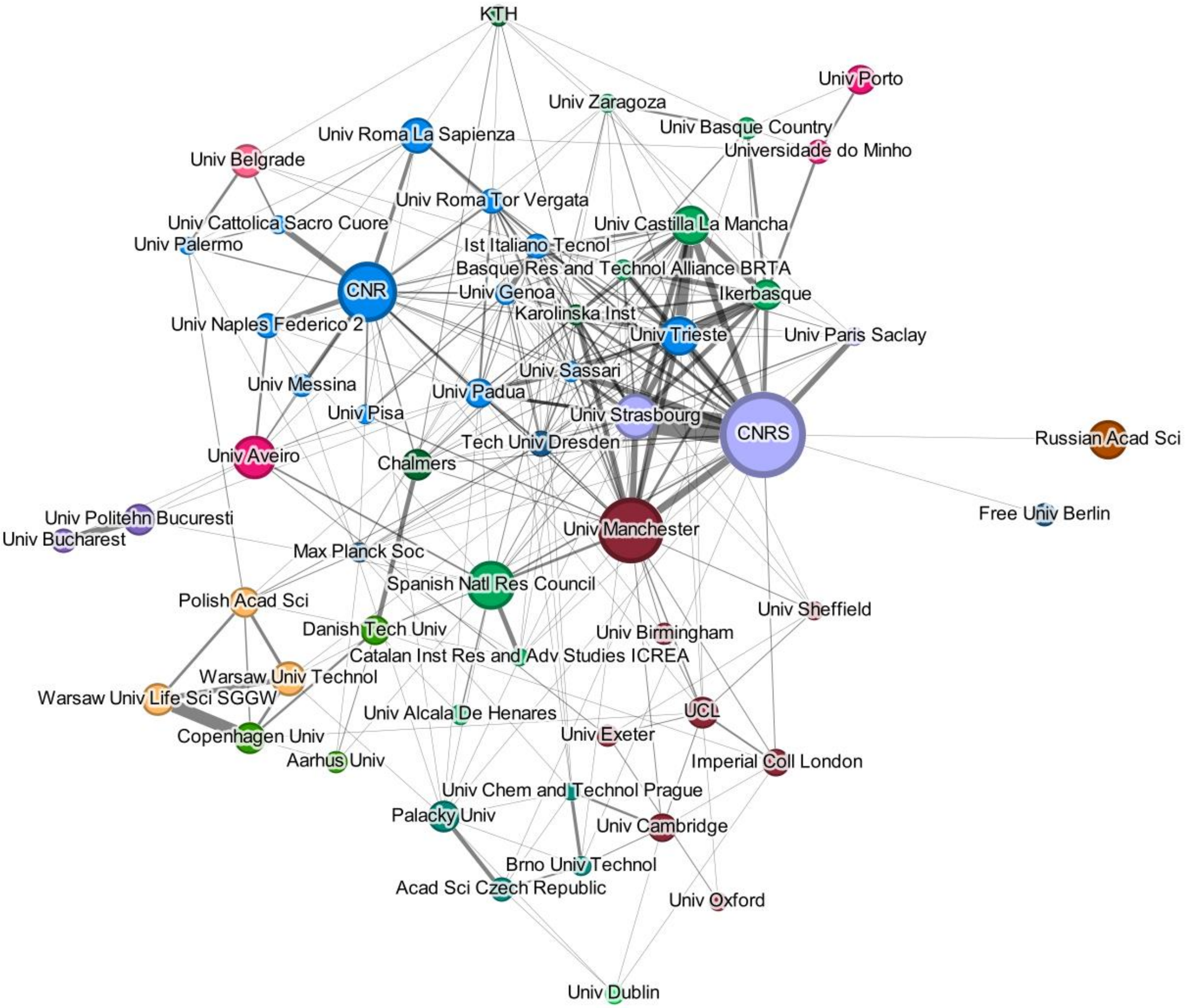

Figure S2. Network of co-publishing organizations in Europe. Publication period: 2008-2024. Note: only organizations with at least 20 publications during this period were included, and organizations which had no co-publications with other organizations were excluded from the analysis. The results were retrieved from © Web of Science 2023 of Clarivate Analytics (UK) Ltd**.**

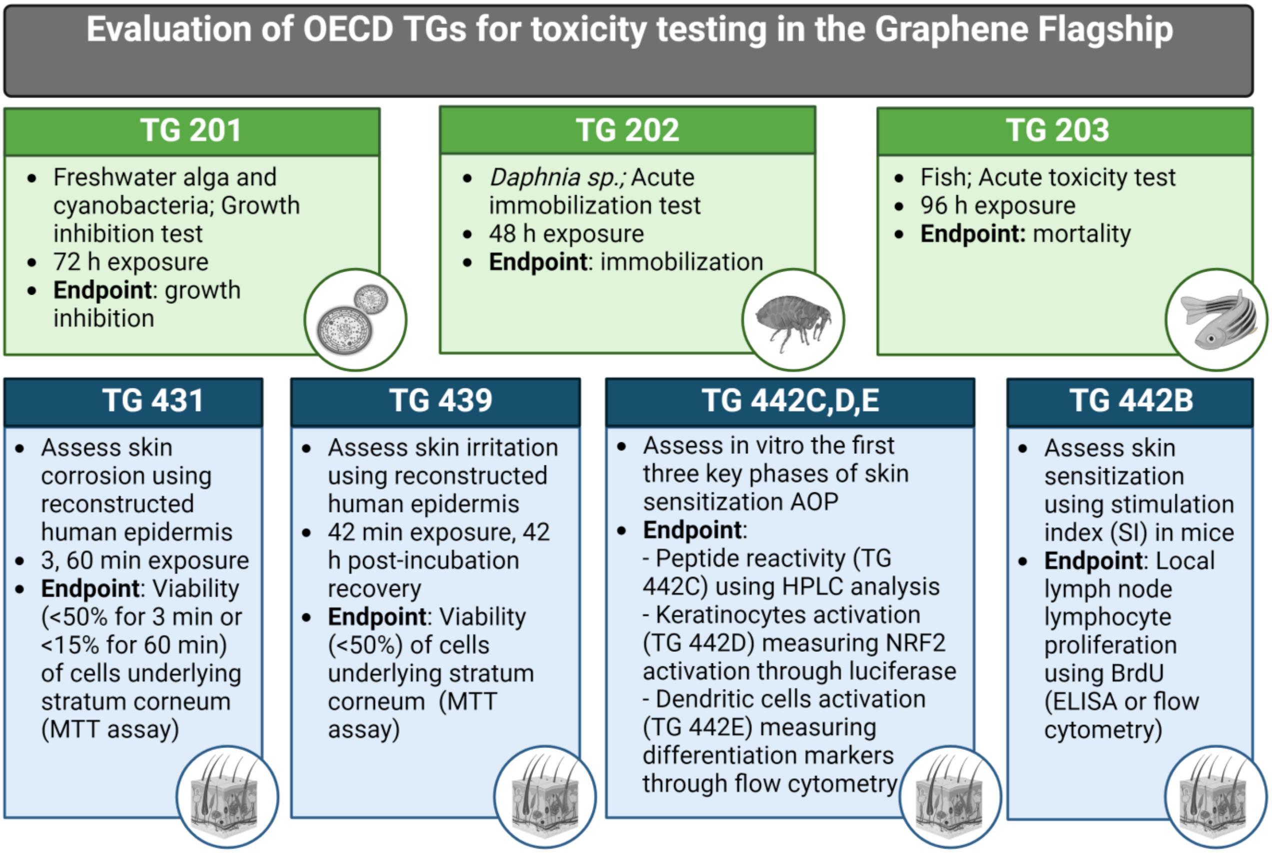

Figure S3. OECD Test Guidelines (TGs). Members of the work package (WP) on Health and Environment of the EU-funded Graphene Flagship have addressed the utility and validity of existing OECD TGs for the testing of chemicals focusing mainly on human skin toxicity and ecotoxicity, and recommendations for modifications of some TGs were provided (refer to main text and references therein). The pictorial elements shown here were created by using BioRender.com.
